# Direct measurement of radiation exposure dose to individual organs during diagnostic computed tomography examination

**DOI:** 10.1038/s41598-021-85060-5

**Published:** 2021-03-08

**Authors:** Kazuta Yamashita, Kosaku Higashino, Hiroaki Hayashi, Kazuki Takegami, Fumio Hayashi, Yoshihiro Tsuruo, Koichi Sairyo

**Affiliations:** 1 Department of Orthopedics, Institute of Biomedical Sciences, Tokushima University Graduate School, 3-18-15 Kuramoto-cho, Tokushima, Tokushima 770-8503 Japan; 2 Department of Pharmaceutical and Health Sciences, Kanazawa University, Kanazawa, Ishikawa Japan; 3 Department of Anatomy, Tokushima University, Tokushima, Tokushima Japan

**Keywords:** Medical research, Oncology, Risk factors, Energy science and technology

## Abstract

Ionizing radiation from Computed tomography (CT) examinations and the associated health risks are growing concerns. The purpose of this study was to directly measure individual organ doses during routine clinical CT scanning protocols and to evaluate how these measurements vary with scanning conditions. Optically stimulated luminescence (OSL) dosimeters were surgically implanted into individual organs of fresh non-embalmed whole-body cadavers. Whole-body, head, chest, and abdomen CT scans were taken of 6 cadavers by simulating common clinical methods. The dosimeters were extracted and the radiation exposure doses for each organ were calculated. Average values were used for analysis. Measured individual organ doses for whole-body routine CT protocol were less than 20 mGy for all organs. The measured doses of surface/shallow organs were higher than those of deep organs under the same irradiation conditions. At the same tube voltage and tube current, all internal organ doses were significantly higher for whole-body scans compared with abdominal scans. This study could provide valuable information on individual organ doses and their trends under various scanning conditions. These data could be referenced and used when considering CT examination in daily clinical situations.

## Introduction

Computed tomography (CT) has many advantages over other imaging modalities because it can be easily performed in a short time and is widely available, allowing physicians to rapidly confirm and/or exclude a diagnosis with higher certainty. Its use has resulted in improved diagnosis and treatment of cancer, trauma, stroke, and cardiac conditions^1,2^. For these reasons, CT has become standard equipment for medical imaging. Furthermore, advances in CT technology have led to new applications and a dramatic increase in its utilization in the past 30 years^3–5^.

A major concern associated with the widespread adoption of CT scanning is increased radiation exposure to patients. Over the past 30 years the average radiation dose to the general public has doubled despite the exposure from natural radiation remaining at nearly the same level^6^.

Data from various national surveys have revealed that CT is a major source of radiation exposure and provides a substantial proportion of the collective dose from medical exposure, for example, approximately 35% in Germany^7^ and 47% in the UK^8^.

The risk to an individual patient is probably small because radiation doses are usually low, but the fact that a large number of people are exposed to medical radiation annually means that even a small individual risk translates into a considerable number of cancer cases. It was estimated that 1.5–2% of cancers in the United States may be attributable to radiation from diagnostic CT^6^.

Many articles have focused on estimating the increased risk of radiogenic cancer incidence as a function of the effective doses from CT scans. Most of these reports have used CT dose index volume and dose length product; these values are preliminarily determined by the manufacturer using standardized 16- and 32-cm phantoms, and are usually displayed on the console when performing CT scans^9–15^. It is known that these dose indicators do not represent absorbed doses of actual patients because they are estimated using phantoms made of synthetic materials with uniform attenuation in two fixed sizes^9^. On the other hand, real human bodies are heterogeneous in terms of tissue composition, and the cross-sectional diameter is highly varied.

To estimate the risk of radiation effects from diagnostic CT scans in individual patients and evaluate the radiation risks appropriately, it is important to know the doses to individual organs in addition to the effective doses. In some studies, organ doses from diagnostic CT were measured in human cadavers^16–18^. However, most of these cadaver studies used embalmed cadavers or only the cadaver torso. The process of embalming using disinfectant/preservative agents, such as formalin, causes remarkable changes to the component elements, so the measured doses may not accurately reflect the doses in the living body. Hence, these studies could provide only limited results.

This report describes the measurement of experimental doses obtained using 6 fresh cadavers, in which multiple point dose measurements were made with high-accuracy dosimeters placed on the skin and inside the cerebrum, thyroid gland, lung, liver, and gonads. Individual organ doses in these fresh cadavers were measured during whole-body, head, chest, and abdominal scans using established clinical protocols. To our knowledge, there are no such prior reports in the literature. The aims of this study were threefold: (1) to directly measure individual organ doses during established clinical CT scanning protocols, (2) to evaluate how the measured values for each organ vary with scanning conditions, (3) to compare the organ doses from the measurement directly with those of the simulated calculation system.

## Materials and methods

Dosimeters were surgically implanted into individual organs of fresh cadavers, including the crystalline lens, thyroid gland, lung, liver, kidney, and gonads. CT scans of cadavers were obtained during various scanning sessions by simulating common clinical methods. After scanning, the dosimeters were extracted, and then the radiation exposure doses for each organ were calculated. And we had simulated each organ doses using a web-based CT dose calculation system (WAZA-ARI v2). This study was approved by the ethics committee of Tokushima University and all methods were performed in accordance with the relevant guidelines and regulations.

### Cadavers

We measured the radiation exposure doses to individual organs in fresh human cadavers. Our institution has a laboratory for cadaver studies, the Tokushima University Clinical Anatomy Education and Research Center, Japan. The cadavers were donated to the laboratory based on patient’s will during their lifetime. In this study, we replicated an established method of actual clinical CT scanning using thawed fresh cadavers that were not embalmed in formalin. Six fresh cadavers (4 male, 2 female; mean height 158.9 [range 146–171] cm; mean body weight 51.6 [range 37.0–64.0] kg) were used in this study.

### Dosimetry

**Figure 1:**
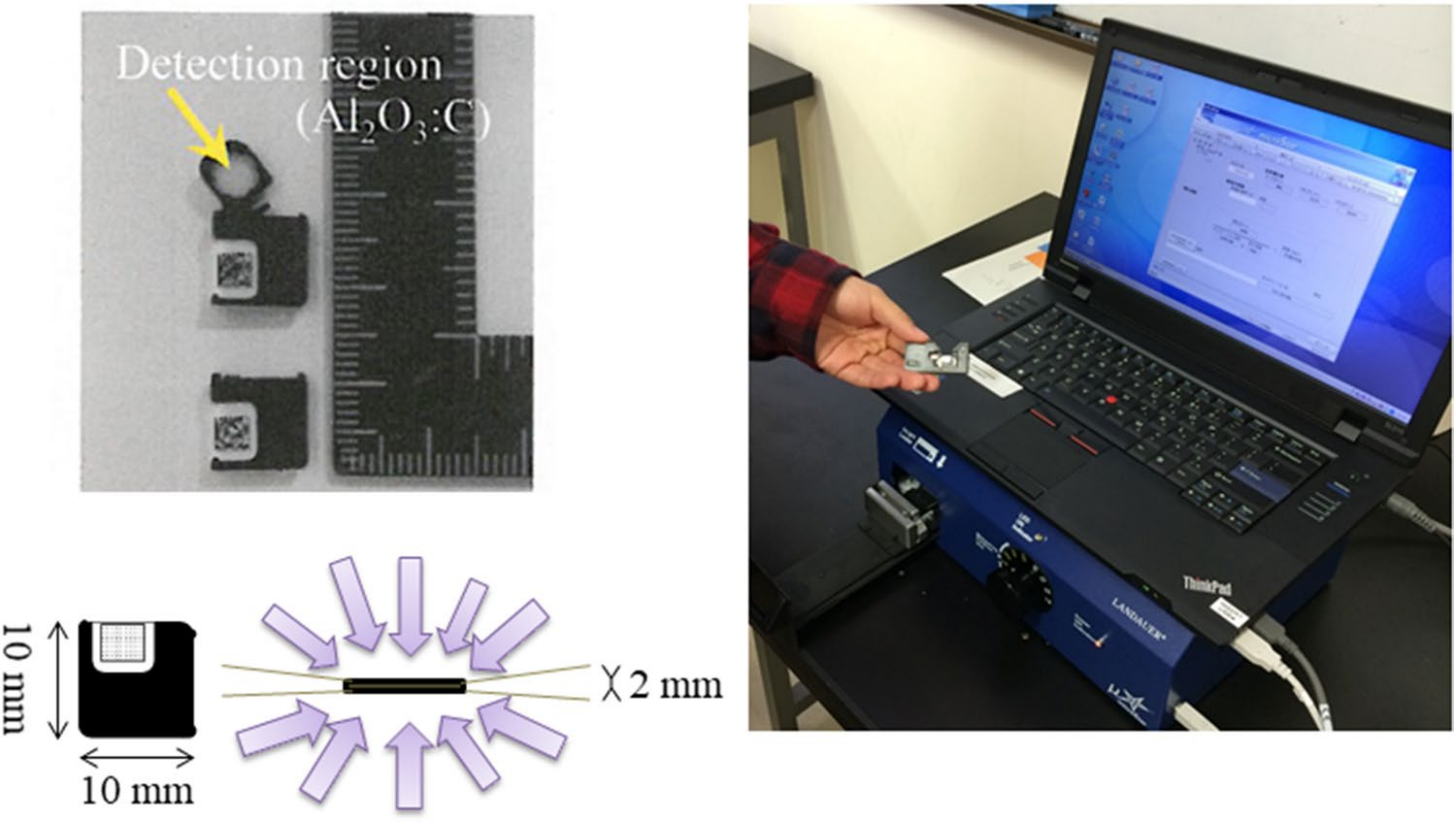
Optically stimulated luminescence (OSL) dosimeter (NanoDot) and reading device (microStar). Advantages of this dosimeter include its small size, light weight, and low detection efficiency. In addition, it does not obscure the obtained image.

We used optically stimulated luminescence (OSL) dosimeters (NanoDot, Landauer, Inc., Glenwood, IL)^19–21^ (Fig. 1). The outer dimensions of each dosimeter were 1 cm × 1 cm × 2 mm, and the detection region is a disk shape measuring 5 mm in diameter and 0.2 mm thickness. The dosimeters use carbon-doped alumina (Al_2_O_3_:C) as a luminescence material, and a similar OSL dosimeter has been widely used for monitoring the personal exposure dose of medical staff because of its long-term stability. Each dosimeter has a bar code on the surface to allow for management of detailed information from individual dosimeters. Dosimeters were stored in a transparent plastic bag to prevent staining of the detection region during use in the study. The main feature of this dosimeter model is its low detection efficiency; that is, the dosimeters do not interfere with the X-ray field when X-rays pass through them^22^. These characteristics are indispensable to achieving the purpose of this study. To reduce the effect of exposure to natural radiation, the NanoDot OSL dosimeters are stored in an annealing machine, with continuous exposure to LED light to initialize them.

### Dosimeter positioning

**Figure 2:**
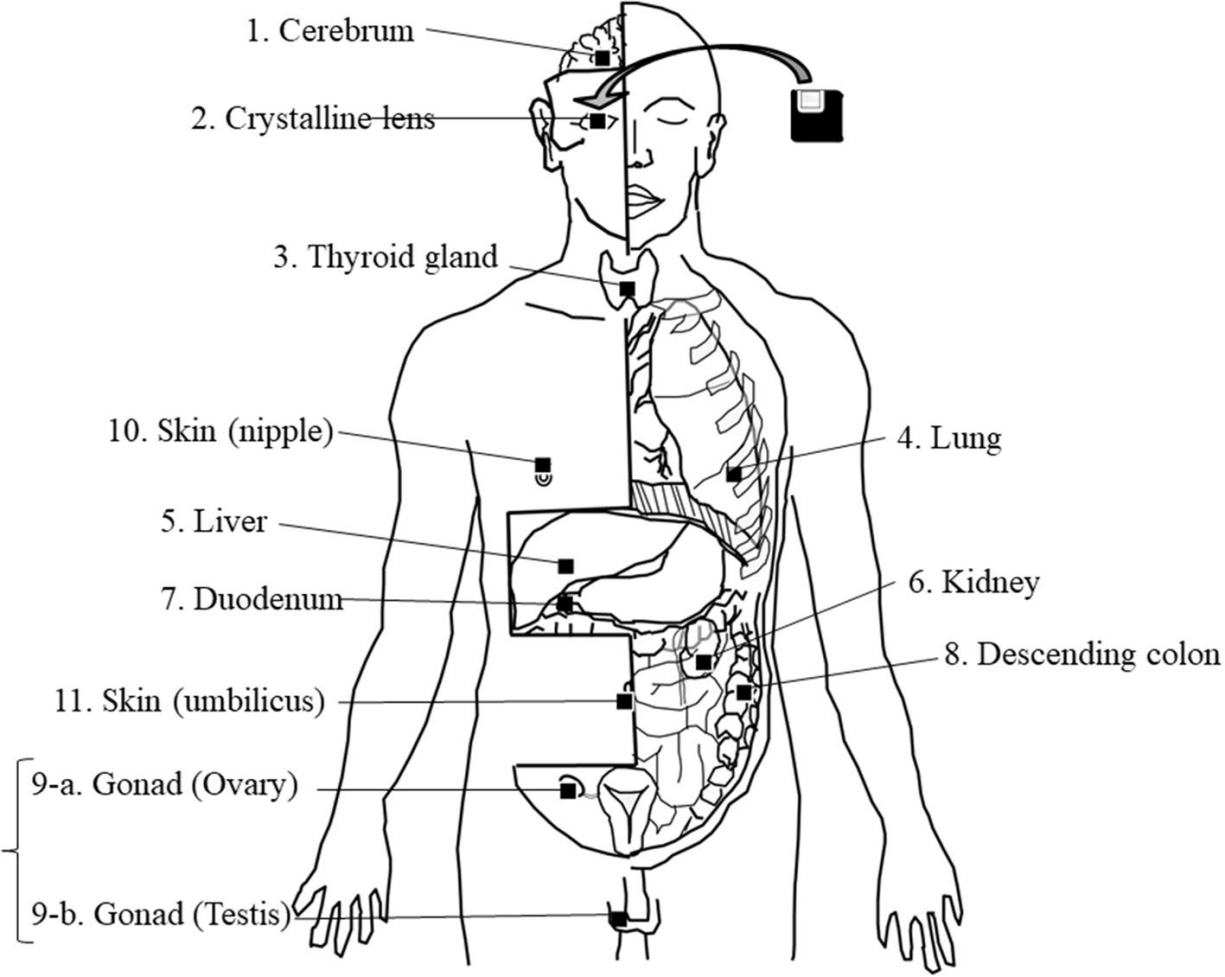
Dosimeter positioning. All dosimeters were implanted and fixed using surgical sutures and tape. In total, 11 dosimeters were used for each whole-body cadaver.

In total, 11 locations were selected for the placement of OSL dosimeters: the right cerebrum, the right crystalline lens, the thyroid gland, left lung, the right lobe of the liver, the left kidney, descending part of the duodenum, the descending colon, the right gonad, and the skin over the right nipple, and the umbilicus. These locations and organs were selected due to the order of priority considering the sensitivity for radiation exposure. The following surgical procedures were performed by 3 surgeons to place the dosimeters (Fig. 2). Using a surgical burr and bone chisel, a square bone window of 2 × 2 cm was made at a point 7 cm from the parietal region of the head on a line joining the parietal region to the right ear. One dosimeter was implanted into the center of the right cerebrum. A vertical 1.5-cm incision was made in the right conjunctiva, and then one dosimeter was implanted just behind the crystalline lens. A horizontal 2-cm incision was made at the inferior horn of the thyroid cartilage, and then one dosimeter was implanted at the center of the thyroid gland. An oblique 2-cm incision was made 15 cm to the left from the center of the sternum and between the 5th and 6th ribs. A sharp-pointed knife and long forceps were used to implant one dosimeter into the inferior lobe of the left lung. Midline vertical and horizontal incisions were made in the anterior abdomen from the xiphoid process to the pubic symphysis. A dosimeter was implanted into the center of the anterior inferior right lobe of the liver and another was implanted into the center of the left kidney. In addition, a dosimeter was placed at the descending part of the duodenum and another was placed in the descending colon. For female cadavers, one dosimeter was placed at the right ovary. For male cadavers, a vertical 2-cm incision was made in the scrotum just above the right testis, and one dosimeter was placed at the right testis. All dosimeters were fixed using surgical sutures and tape. The skull bone was repositioned and all incisions were closed using surgical sutures. A dosimeter was fixed at the left nipple and another at the umbilicus using surgical sutures. All dosimeters were covered with thin nylon film for protection against cadaveric fluids.

### Instrumentation

**Figure 3:**
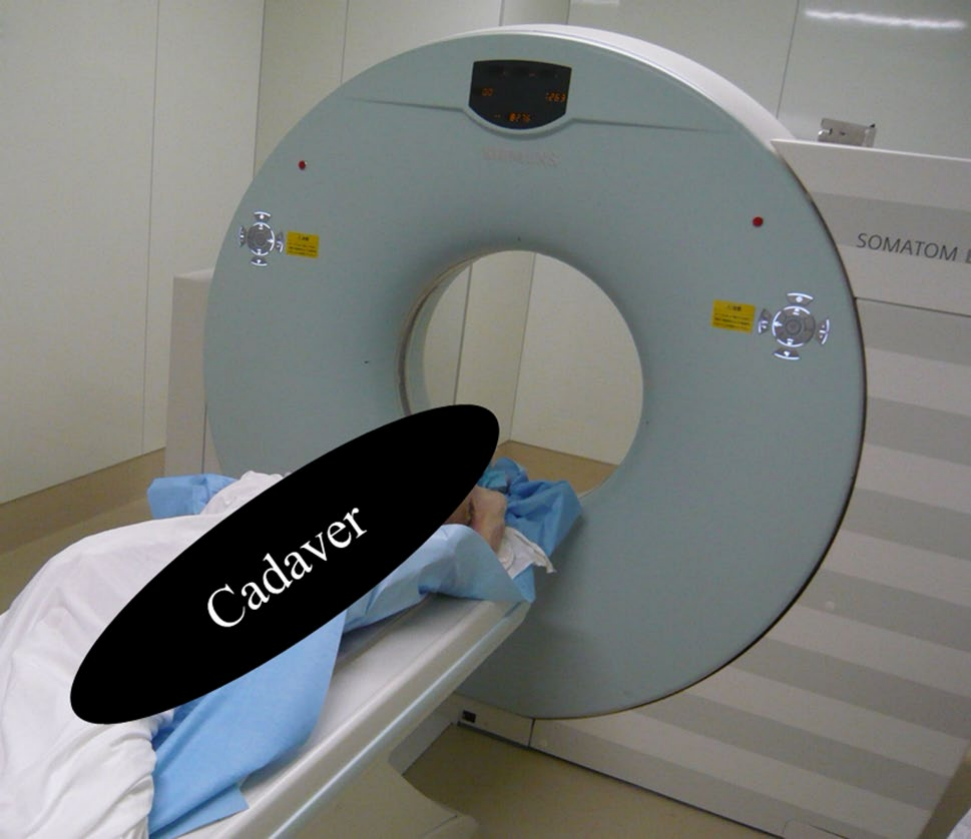
SOMATOM Emotion 16-slice computed tomography scanner (SIEMENS) installed in the cadaver research institution.

All radiation exposures to individual organs of the cadavers were examined using a 16-slice multidetector CT scanner (SOMATOM Emotion 16; Siemens Healthineers, Erlangen, Germany) installed at the cadaver research institution (Fig. 3). The CT scanner is examined as a periodical semiannual inspection, including gantry, collimator and detector by specialists of Siemens company.

### CT settings

**Figure 4:**
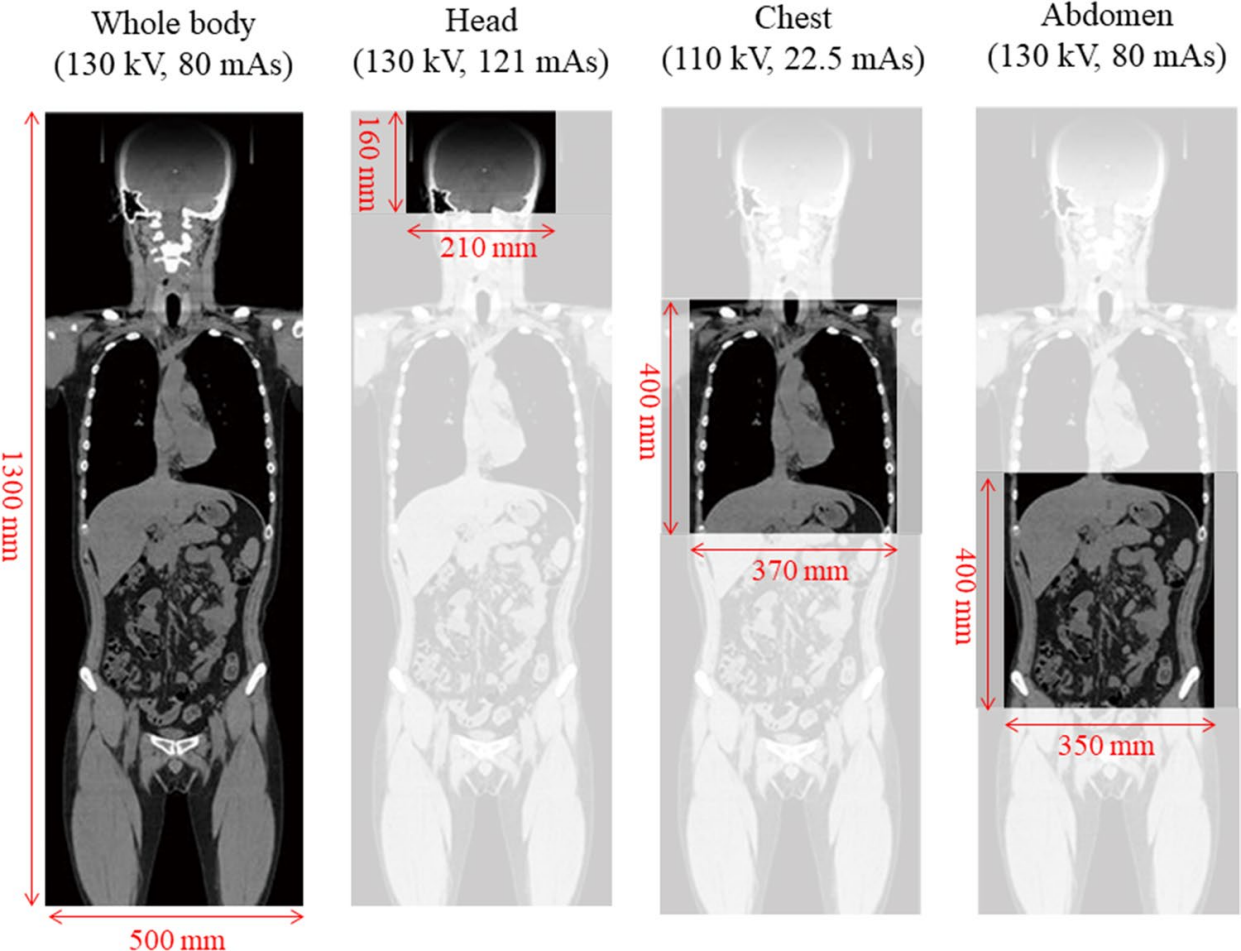
CT examination procedure and irradiation conditions. All examinations were performed using routine clinical protocols.

**Figure 5:**
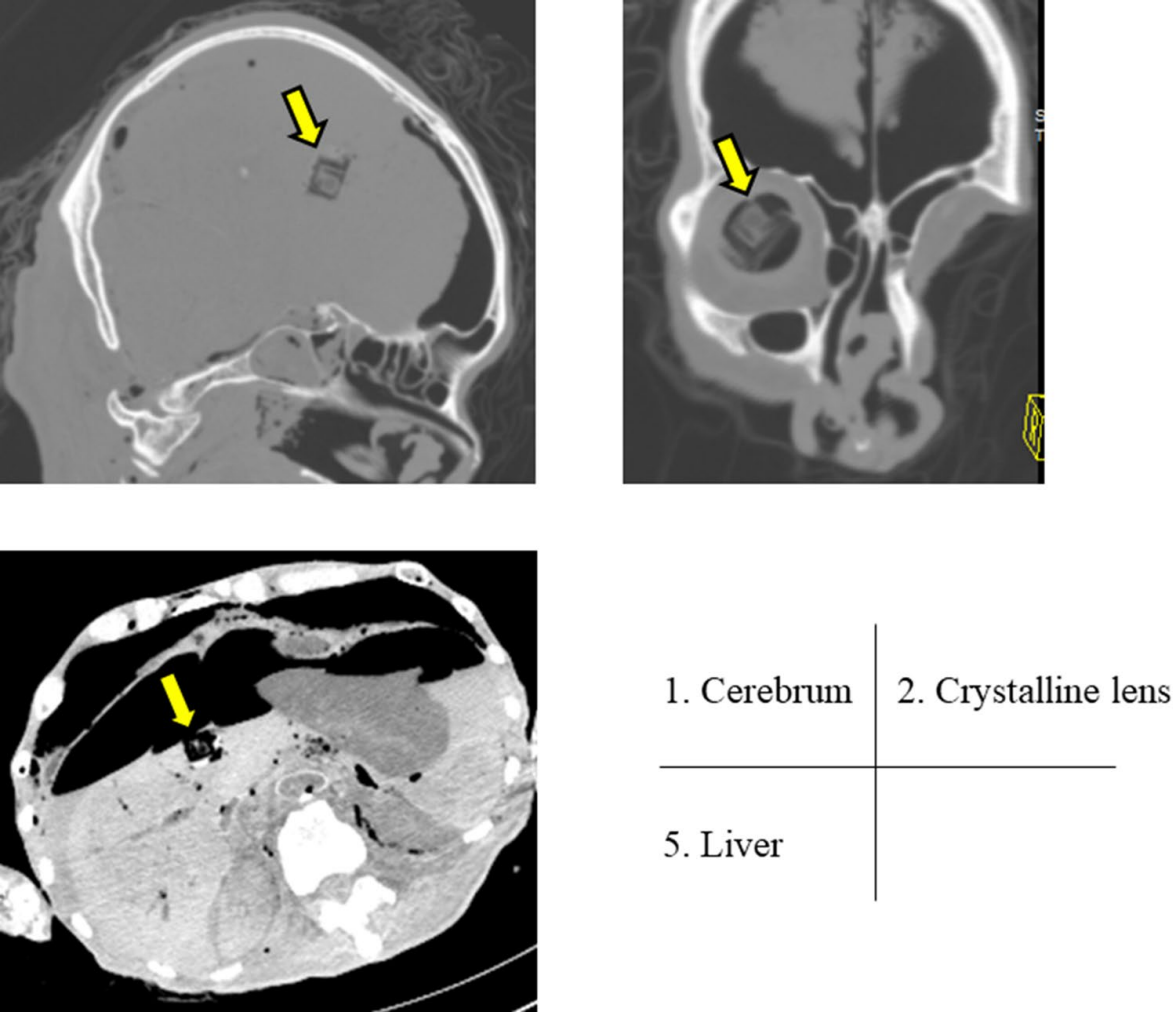
CT imaging of individual organs. CT image showing that all dosimeters (arrow) were placed at appropriate positions and did not affect the image.

We measured radiation exposure doses to individual organs during whole-body, head, chest, and abdominal CT scans. In this study, we selected the scanning protocols widely used in routine practice, which is used in clinical practice. Table 1 and Fig. 4 shows irradiation conditions for whole-body scan, head scan, chest scan and abdomen scan. For scanning, each cadaver was placed on the CT table in the supine position and centered on the x and y axes. Two researchers confirmed that the cadaver was placed in the appropriate centering position. The cadaver was kept in the original position on the scanning bed during the entire scanning procedure. We confirmed that all dosimeters were placed appropriately at each site using the obtained images (Fig. 5).

**Table 1:** Irradiation conditions of four CT scan protocols.

	Whole-body scan	Head scan	Chest scan	Abdominal scan
Tube voltage (kV)	130	130	110	130
Tube current (mAs)	80	121	22.5	80
Pitch factor	0.8	0.55	1.5	0.8
Beam collimation (mm)	16 × 0.6	16 × 0.6	16 × 0.6	16 × 1.2
Rotation time (second)	0.6	1.5	0.6	0.6
Field of view (mm × mm)	1300 × 500	160 × 210	400 × 370	400 × 350

Each irradiation conditions were based on the routine mode of each CT protocol.

### Analysis of radiation exposure

The exposure dose measured using the NanoDot OSL dosimeters was analyzed with a commercial dosimeter reading device (MicroStar, Landauer). The reading device showed that the difference in detection efficiency of the dosimeters was properly accounted for; efficiency was determined by the manufacturer at the time of shipment and recorded in the bar code. Therefore, the absorbed dose can be analyzed using a commonly determined dose calibration factor, which was configured in the settings of the dosimeter reading device. It is known that the effective energy of X-rays used in CT scans is higher than that for the general X-ray diagnosis, so in principle, it is preferred to perform dose calibration taken into consideration the quality of X-rays. However, because the mixing ratio of direct and scattered X-rays differs depending on the organ placement under the usage conditions in this study, it is difficult to determine the effective energy of the X-ray field inside the human body exactly. According to the previous research^19^, it has shown that the uncertainty is at most 15% when the manufacturer-determined calibration factor is used to analyze doses using X-rays at other tube voltages. Furthermore, a study has been reported in which the dose determined by the OSL dosimeter using this method shows the correct entrance surface dose during CT examination within the margin of error^20^. Based on these facts, we used the manufacturer-determined calibration factor to analyze the absorbed dose from CT examinations. The systematic uncertainty (15%) related to this method is much smaller than the error introduced by differences between patients.

### Organ doses simulation using web-based CT dose calculation system

In order to compare the experimental results with reference values, we calculated the organ doses in each CT scanning using a web-based CT dose calculation system (WAZA-ARI v2)^23–25^. This is a dose calculation system in which results of the Monte-Carlo simulation calculated by a supercomputer were databased, and we can estimate the organ doses according to the actual scan parameters applied in the CT equipment of each company based on various standard-type human body models. Standard female body size (155 cm, 52 kg, BMI 21.6) was chosen for the phantom because the mean height and mean body weight we used for the organ dose measurement were 158.9 cm, 51.6 kg, respectively. After substitution of the scanner model and each irradiation condition, organ doses were calculated.

### Statistical analysis

The doses for each organ and CT scanning procedure were compared using the paired t-test (SPSS software 11.0J; SPSS Inc., Tokyo, Japan). The doses of comparison were below, (1) the doses at deep organ and shallow organ in whole-body scan, (2) the doses at two location of skin in whole-body scan, (3) the doses of internal organs in whole-body scan and in abdominal scan. A *p* value < 0.05 was considered to indicate statistical significance.

## Results

Average values of individual organ doses for the 6 fresh cadavers are shown in Table 2. These result from the following two phenomena: the direct exposure dose to each organ (e.g., the cerebrum, crystalline lens, thyroid gland, lung, testis, and nipple skin in whole-body scans), and the scatter exposure dose to each organ (e.g., liver, kidney, duodenum, descending colon, ovary, and umbilicus in head scans).

**Table 2:** Average of individual organ dose measurements using each CT scan procedure.

n = 6 (mGy)	Whole-body scan**	Head scan	Chest scan	Abdominal scan**	*P* value** (paired t test)
1. Cerebrum	14.33 ± 1.30	31.18 ± 2.18	0.02 ± 0.01	0.06 ± 0.05	< 0.001
2. Crystalline lens	13.22 ± 3.70	29.95 ± 3.84	0.03 ± 0.01	0.13 ± 0.12	< 0.001
3. Thyroid gland	19.57 ± 2.67^a^	1.32 ± 0.70	2.14 ± 0.32	0.25 ± 0.13	< 0.001
4. Lung	15.53 ± 1.59	0.76 ± 0.90	1.91 ± 0.23	2.88 ± 2.85	< 0.001
5. Liver	15.68 ± 1.08	0.13 ± 0.09	1.62 ± 0.61	12.01 ± 2.02	0.008
6. Kidney	12.04 ± 1.96^a^	0.04 ± 0.03	0.44 ± 0.38	9.94 ± 1.32	0.014
7. Duodenum	17.58 ± 1.18	0.05 ± 0.03	0.13 ± 0.06	12.82 ± 1.36	< 0.001
8. Descending colon	14.56 ± 3.47	0.05 ± 0.03	0.22 ± 0.23	10.35 ± 2.20	0.007
9-a. Gonad (testis) (n = 4)	15.62 ± 0.99	0.01 ± 0.0004	0.01 ± 0.002	0.56 ± 0.34	< 0.001
9-b. Gonad (ovary) (n = 2)	14.26 ± 1.84	0.05 ± 0.03	0.08 ± 0.005	11.96 ± 0.46	< 0.001
10. Skin (nipple)	13.97 ± 1.91^b^	0.40 ± 0.23	1.63 ± 0.18	5.50 ± 4.73	< 0.001
11. Skin (umbilicus)	19.44 ± 3.62^a,b^	0.07 ± 0.04	0.14 ± 0.07	15.63 ± 2.09	0.007

^a^Measured doses to shallow/surface organs (e.g., thyroid gland and umbilical skin) were higher than those of deep organs (e.g., kidney) under the same irradiation conditions (130 kV, 80 mAs) (*p* = 0.06).

^b^The dosimeters at both the nipple and the umbilicus were on the skin surface, but the measured dose of umbilicus was higher in whole-body scans (*p* = 0.09).

**All measurement values were significantly higher in whole-body scans than in abdominal scan under the same scanning condition (130 kV, 80 mAs) (*p* < 0.05).

For whole-body scans, the average values of individual organ doses were as follows: cerebrum 14.33 mGy, crystalline lens 13.22 mGy, thyroid gland 19.57 mGy, lung 15.53 mGy, liver 15.68 mGy, kidney 12.04 mGy, duodenum 17.58 mGy, descending colon 14.56 mGy, gonad (testis 15.62 mGy, ovary 14.26 mGy), nipple skin 13.97 mGy, and umbilical skin 19.44 mGy (Table 2). The measured doses of surface and shallow organs (e.g., thyroid gland and skin) were higher than those of deep organs (e.g., kidney), during CT scanning with the same irradiation conditions (130 kV, 80 mAs) (Table 2a). The measurement doses at the umbilicus were higher than those at the nipple, even though both were located on the skin surface (Table 2b).

For head scans, the average values of individual organ doses were as follows: cerebrum 31.18 mGy, crystalline lens 29.95 mGy, thyroid gland 1.32 mGy, lung 0.76 mGy, liver 0.13 mGy, kidney 0.04 mGy, duodenum 0.05 mGy, descending colon 0.05 mGy, gonad (testis 0.01 mGy, ovary 0.05 mGy), nipple skin 0.4 mGy, and umbilical skin 0.07 mGy (Table 2). The measured doses in the cerebrum were higher for head scans than for whole-body scans because of differences in the exposure time product (tube current-time product × scanning time; 121 mAs in head scans and 80 mAs in whole-body scans). The measured dose of the crystalline lens, which was within the irradiated area, was remarkably high, and the measured value of thyroid grand, which was close to but not within the irradiated area, was relatively high.

For chest scans, the average values of individual organ doses were as follows: cerebrum 0.02 mGy, crystalline lens 0.03 mGy, thyroid gland 2.14 mGy, lung 1.91 mGy, liver 1.62 mGy, kidney 0.44 mGy, duodenum 0.13 mGy, descending colon 0.22 mGy, gonad (testis 0.01 mGy, ovary 0.08 mGy), nipple skin 1.63 mGy, and umbilical skin 0.14 mGy (Table 2). In this case, although the dosimeters were placed in the directly irradiated area, the measured doses of each organ in the chest region were not so high because both the tube voltage and tube current were relatively low (110 kV, 22.5 mAs) compared with the other scanning conditions.

For abdominal scans, the average values of individual organ doses were as follows: cerebrum 0.06 mGy, crystalline lens 0.13 mGy, thyroid gland 0.25 mGy, lung 2.88 mGy, liver 12.01 mGy, kidney 9.94 mGy, duodenum 12.82 mGy, descending colon 10.35 mGy, gonad (testis 0.56 mGy, ovary 11.96 mGy), nipple skin 5.5 mGy, and umbilical skin 15.63 mGy (Table 2). The measured doses of shallow organs (e.g., the duodenum) were higher than those of deep organs (e.g., the kidney and descending colon) (Table 2). Measured values of the ovary were higher than those of the testis because the ovary was located in the irradiated area and the testis was not.

Table 3 shows the simulated organ doses, CTDI (computed tomography dose index) vol and DLP (dose length product) using web-based CT dose calculation system, WAZA-ARI v2. CTDI vol and DLP of each CT scan protocol were 13.33 mGy, 1732.35  mGy cm in whole-body scan, 63.67 mGy, 1018.74  mGy cm in head scan, 1.31 mGy, 52.38 mGy cm in chest scan, 13.36 mGy, 534.23 mGy cm in abdominal scan, respectively. Simulated organ doses in each CT scans were relatively higher than direct measured organ doses (Tables 2, 3).

**Table 3:** Simulated organ doses, CTDI (computed tomography dose index) vol and DLP (dose length product) during each CT scan procedure using web-based CT dose calculation system.

	Whole-body scan	Head scan	Chest scan	Abdominal scan
1. Cerebrum	23.47	50.1	0.03	0.01
2. Crystalline lens	23.65	50.76	0.03	0.02
3. Thyroid gland	40.7	2	3.79	0.3
4. Lung	25.44	0.26	2.43	7.09
5. Liver	25.47	0.04	2.27	23.37
6. Kidney	27.3	0.02	2.11	26.5
7. Duodenum	24.79	0	0.21	24.3
8. Descending colon	24.77	0	0.17	24.17
9. Gonad	20.33	0	0.01	18.48
10. Skin	17.2	0.07	2.43	3.76
CTDI vol (mGy)	13.33	63.67	1.31	13.36
DLP (mGy cm)	1732.35	1018.74	52.38	534.49

## Discussion

In this study, we directly measured individual organ doses during CT examinations using non-embalmed fresh whole-body cadavers. Dosimeters were implanted into individual organs and doses were calculated after various CT examinations. This study revealed accurate and reliable organ dose data for each protocol of diagnostic CT examination. Higher doses were observed on CT scanning with higher tube voltage and tube current. On the other hand, compared with the measured doses of abdominal scanning, all measured values of whole-body scans were significantly higher, although the tube voltage and tube current were the same (130 kV, 80 mAs) (Table 2**).

Previously, limited knowledge of these radiation exposure doses was available despite their importance in estimating the risk posed by CT examinations. Numerous studies have used anthropomorphic and/or cylindrical acrylic phantoms to estimate radiation exposure doses for individual organs during CT examinations; however, other studies reported significant variation according to the type of simulation software^26^. Other studies have directly measured CT organ doses in human subjects. Padole et al. compared directly measured and estimated organ doses obtained from commercial dose calculation software^27^. They reported that most organ doses estimated from commercial dose calculation software were significantly greater than directly measured organ doses. We also compared directly measured and estimated organ doses and our results showed same tendency. Regarding to these differences, Padole et al. described that differences may be attributed to the “entire organ” dose estimation with software compared to the “point organ” dose measurements with dosimeters^27^. Additionally, it should be pretty deformative for each organ due to the implantation procedure and that is why the differences had occurred, though the measurement of each organ doses using multiple dosimeters could have thought the real organ doses. Griglock et al. measured organ doses directly using OSL dosimeters in embalmed cadavers and stated that the anatomic and material composition of cadavers is comparable to that of living patients^16^. Their results indicated that doses to deep organs were lower than those to surface and shallow organs. Our results also showed that doses in deep organs (e.g., the kidney and descending colon) were lower than those of surface and shallow organs (e.g., the skin and thyroid gland) (Table 2a, *p* = 0.06).

We found that all internal organ doses, such as those to the liver and kidney, were significantly higher in whole-body scans compared with abdominal scans at the same tube voltage and tube current (Table 2**, *p* = 0.09). The differences between these two CT examination protocols were the beam collimation and the field of view. The presence of scatter radiation due to irradiation from head to chest might cause increased abdominal doses to the internal organs. Advances in the development of CT scanners have made it easy to obtain whole-body CT scans. In addition, CT scans can be performed within seconds and do not require sedation of very young patients, in contrast to magnetic resonance imaging. Furthermore, compared with other imaging modalities, CT examinations do not strongly depend on the skill of the operator. In an emergency, a whole-body CT might be ordered even if there are no abdominal symptoms. On the other hand, if there are no abnormalities in the chest and a CT scan of the abdominal area is needed, the irradiation field should be limited to only the abdomen. Then, the organ doses due to direct irradiation to the chest as well as the doses due to scatter irradiation can be reduced.

In whole-body scan, the measured doses at the umbilicus were significantly higher than those of the nipple, even though both are located on the body surface (Table 2b). This difference might be attributable to scatter radiation. There are several parenchymal organs in the abdomen, such as liver, kidney, and pancreas; X-ray irradiated onto these organs are scattered X inside the body. Several studies have reported simulated organ doses using anthropomorphic phantoms constructed from materials that mimic the radiation attenuation characteristics of human tissue^28–30^. However, these studies using anthropomorphic phantoms had some difficulties because the phantoms had physical limitations: the constituents for simulating parenchymal and hollow viscus organs and soft tissue are completely different from the actual structures in the human body. In this study, we used fresh cadavers rather than embalmed cadavers or anthropomorphic phantoms. Consequently, the measured accuracy and reliability of organ doses were relatively higher than those reported in previous studies.

This study revealed the influence of scattered X-rays on organs that were outside the irradiated field. For head scans, the exposure doses to the thyroid gland, ovary, and nipple skin were 1.32 mGy, 0.05 mGy, and 0.4 mGy, respectively. For the abdomen, the exposure doses to the thyroid gland, lung, and nipple skin were 0.25 mGy, 2.88 mGy, and 5.5 mGy, respectively; these seem to be relatively high. Special attention should be given to individuals who have undergone CT scans multiple times or at a young age. A study of 180,000 young people undergoing CT scans in the United Kingdom found an increasing risk of leukemia and brain cancer with increasing radiation dose^31^. Another study had shown that cancer incidence was increased by 24% among 680,000 Australians who had undergone CT scans between ages 0–19 years compared with the incidence in over 10 million unexposed persons^32^. Incidence rates were increased for most types of solid cancer, as well as leukemia and brain cancer. However, some radiation experts have questioned the validity of earlier indirect estimates based on projected risks^33,34^. Thus, much uncertainty currently remains as to whether there is any definitive cancer risk from CT scan exposure. ICRP adopted the Linear non-threshold model (LNT model) in the radiological protection system, but the concept of safety threshold doses cannot be applied for the patients in CT examinations considering carcinogenesis and chromosomal abnormalities as stochastic effects^35^. In any case, shielding from scatter radiation during CT scan exposure might be considered especially for radiosensitive organs, such as the crystalline lens, thyroid gland, and gonads.

We have demonstrated the feasibility of measuring organ doses in non-embalmed fresh human cadavers using scanning protocols from routine clinical practice. The findings of this study could provide valuable information on measured organ doses and their trends under various scanning conditions.

The major strength of this study is that we measured individual organ doses using 6 whole-body non-embalmed fresh cadavers and OSL dosimeters. Another strength is that we comprehensively obtained measurements in 4 CT scanning protocols: whole-body, head, chest, and abdominal scans. The measured doses reflect actual organ doses received by the tissues of patients undergoing clinical CT examination.

Our study has some limitations. First, doses for individual organs were measured in 6 cadavers, but the body size of the cadavers was relatively small for adults. All cadavers were elderly and did not include any children. Thus, these results may not be applicable to patients with larger body size or to children. Compared with adults, children are reported to possibly receive higher organ doses from comparable investigations as their bodies are much smaller and the shielding of individual organs is lower^36^. The cancer risk in children due to radiation exposure from CT scanning is much greater than that in adults^32^, so further research is warranted. Second, we used only one type of multidetector-row CT scanner for measurements; hence, the results may not be applicable to other CT scanners. Third, we measured individual organ doses using only routine clinical protocols and did not obtain measurements using dose reduction techniques, such as low-dose protocols and automatic exposure control.

In conclusion, this study demonstrated measurements of organ doses from various clinical CT examinations using fresh non-embalmed human cadavers. The complexity of the variation in individual organ doses was investigated in detail using OSL dosimeters. These data could be referenced and used when considering CT examination in daily clinical situations.

## Data Availability

The datasets used and/or analysed in this study are available from the corresponding author on reasonable request.
