# Author Correction: Direct measurement of radiation exposure dose to individual organs during diagnostic computed tomography examination

**DOI:** 10.1038/s41598-022-08891-w

**Published:** 2022-03-23

**Authors:** Kazuta Yamashita, Kosaku Higashino, Hiroaki Hayashi, Kazuki Takegami, Fumio Hayashi, Yoshihiro Tsuruo, Koichi Sairyo

**Affiliations:** 1 Department of Orthopedics, Institute of Biomedical Sciences, Tokushima University Graduate School, Tokushima, Tokushima Japan; 2 Department of Pharmaceutical and Health Sciences, Kanazawa University, Kanazawa, Ishikawa, Japan; 3 Department of Anatomy, Tokushima University, Tokushima, Tokushima, Japan

Correction to: *Scientific Reports* 10.1038/s41598-021-85060-5, published online 08 March 2021

The original version of this Article contained errors.

The Siemens scanner used in this study provides “effective mAs” in the console, while other scanners used by the Authors displayed “mAs” value. In the original study the Authors incorrectly used “effective mAs” as “mAs” for the simulation, therefore all simulation results were not correct. The Authors now re-checked all the calculation parameters used in the simulation in detail and redid all simulations. This results in the following changes to the Article.

In Results,

“The measured doses of surface and shallow organs (e.g., thyroid gland and skin) were higher than those of deep organs (e.g., kidney), during CT scanning with the same irradiation conditions (130 kV, 100 mAs) (Table 2a).”

now reads

“The measured doses of surface and shallow organs (e.g., thyroid gland and skin) were higher than those of deep organs (e.g., kidney), during CT scanning with the same irradiation conditions (130 kV, 80 mAs) (Table 2a).”

In the next paragraph,

“The measured doses in the cerebrum were higher for head scans than for whole-body scans because of differences in the exposure time product (tube current-time product × scanning time; 220 mAs in head scans and 100 mAs in whole-body scans).”

now reads

“The measured doses in the cerebrum were higher for head scans than for whole-body scans because of differences in the exposure time product (tube current-time product × scanning time; 121 mAs in head scans and 80 mAs in whole-body scans).”

and then,

“In this case, although the dosimeters were placed in the directly irradiated area, the measured doses of each organ in the chest region were not so high because both the tube voltage and tube current were relatively low (110 kV, 15 mAs) compared with the other scanning conditions.”

now reads

“In this case, although the dosimeters were placed in the directly irradiated area, the measured doses of each organ in the chest region were not so high because both the tube voltage and tube current were relatively low (110 kV, 22.5 mAs) compared with the other scanning conditions.”

In the last paragraph of Results,

“Table 3 shows the simulated organ doses, CTDI (computed tomography dose index) vol and DLP (dose length product) using web-based CT dose calculation system, WAZA-ARI v2. CTDI vol and DLP of each CT scan protocol were 12.53 mGy, 1270.28 mGy cm in whole-body scan, 58.64 mGy, 331.34 mGy cm in head scan, 1.22 mGy, 39.01 mGy cm in chest scan, 11.23 mGy, 258.89 mGy cm in abdominal scan, respectively. Simulated organ doses in each CT scans were relatively higher than direct measured organ doses (Table 2, 3).”

now reads

“Table 3 shows the simulated organ doses, CTDI (computed tomography dose index) vol and DLP (dose length product) using web-based CT dose calculation system, WAZA-ARI v2. CTDI vol and DLP of each CT scan protocol were 13.33 mGy, 1732.35 mGy cm in whole-body scan, 63.67 mGy, 1018.74 mGy cm in head scan, 1.31 mGy, 52.38 mGy cm in chest scan, 13.36 mGy, 534.23 mGy cm in abdominal scan, respectively. Simulated organ doses in each CT scans were relatively higher than direct measured organ doses (Table 2, 3).”

In Discussion,

“On the other hand, compared with the measured doses of abdominal scanning, all measured values of whole-body scans were significantly higher, although the tube voltage and tube current-time product were the same (130 kV, 100 mAs) (Table 2**).”

now reads

“On the other hand, compared with the measured doses of abdominal scanning, all measured values of whole-body scans were significantly higher, although the tube voltage and tube current-time product were the same (130 kV, 80 mAs) (Table 2**).”

In Table 1 the tube current (mAs) parameters: 100 mAs for whole-body scan, 220 mAs for head scan, 15 mAs for chest scan and 100 mAs for abdominal scan now read 80, 121, 22.5, and 80, respectively.

In the caption of Table 2,

“Measured doses to shallow/surface organs (e.g., thyroid gland and umbilical skin) were higher than those of deep organs (e.g., kidney) under the same irradiation conditions (130 kV, 100 mAs) (p=0.06).”

now reads

“Measured doses to shallow/surface organs (e.g., thyroid gland and umbilical skin) were higher than those of deep organs (e.g., kidney) under the same irradiation conditions (130 kV, 80 mAs) (p=0.06).”

and

“All measurement values were significantly higher in whole-body scans than in abdominal scan under the same scanning condition (130 kV, 100 mAs) (*p* < 0.05).”

now reads

“All measurement values were significantly higher in whole-body scans than in abdominal scan under the same scanning condition (130 kV, 80 mAs) (*p* < 0.05).”

All results in Table 3 are revised. The original Table 3 is listed below for the record.

**Table 3:** Simulated organ doses, CTDI (computed tomography dose index) vol and DLP (dose length product) during each CT scan procedure using web-based CT dose calculation system.

	Whole-body scan	Head scan	Chest scan	Abdominal scan
1. Cerebrum	17.5	123.31	0.01	< 0.01
2. Crystalline lens	16.15	117.2	0.01	0.01
3. Thyroid gland	26.56	0.8	1.25	0.23
4. Lung	16.92	0.16	0.83	4.55
5. Liver	17.18	0.03	0.7	14.05
6. Kidney	16.72	0.01	0.27	14.64
7. Duodenum	17.99	< 0.01	0.06	16.02
8. Descending colon	17.79	< 0.01	0.09	15.73
9. Gonad	23.34	< 0.01	< 0.01	8.57
10. Skin	17.9	0.12	0.19	3.92
CTDI vol (mGy)	12.53	58.64	1.22	11.23
DLP (mGy cm)	1270.28	331.34	39.01	258.89

Finally, Figure 4 was updated to reflect the correct scanning parameters.

The original Article has been corrected.

